# Functional redundancy of R2R3-MYB transcription factors involved in anthocyanin biosynthesis is manifested in anther pigmentation in petunia

**DOI:** 10.5511/plantbiotechnology.23.1120a

**Published:** 2024-03-25

**Authors:** Mashiro Yuhazu, Ryoko Hara, Mei Kimura, Akira Kanazawa

**Affiliations:** 1 Research Faculty of Agriculture, Hokkaido University, Sapporo, Hokkaido 060-8589, Japan

**Keywords:** anther pigmentation, anthocyanin biosynthesis, functional redundancy, *Petunia hybrida*, transcription factors

## Abstract

Accumulation of anthocyanin provides pigmentation in plant tissues. In petunia, gene expression profiles that lead to anthocyanin production have been extensively characterized in terms of pigmentation in flower petals. Anthers are also pigmented, but the transcriptional control of the genes for anthocyanin biosynthesis in anthers has not been fully characterized. Here we addressed this issue by analyzing the expression of structural genes and genes encoding transcription factors (TFs) of the pathway. Ectopic expression of the *PURPLE HAZE* (*PHZ*) gene encoding an R2R3-MYB activator induced pigmentation in anthers. The pigmentation was accompanied by an increase in mRNA levels of *AN1*, *MYB27* and *MYBx* among the genes encoding TFs. Among the structural genes, mRNA levels of four late biosynthetic genes (LBGs) were higher in the transformants than in the wild type. Analyses of gene expression profile using commercial varieties indicated that mRNA levels of *MYB27*, *MYBx* and LBGs and of *AN4*, responsible for anther pigmentation, were higher in pigmented anthers than in nonpigmented. Differences in the gene expression profile between pigmented anthers induced by ectopic *PHZ* expression and their nonpigmented control and those between pigmented anthers and nonpigmented anthers of existing varieties were thus remarkably similar. These observations suggest that a high level of expression of the LBGs is characteristic of pigmented anthers and that ectopic *PHZ* expression in the *an4*^−^ genetic background induced changes in the transcriptional network toward the state established in pigmented anthers, which is intrinsically brought about by the function of *AN4*.

## Introduction

Anthocyanins are a group of flavonoids that accumulate in plant cells, providing purple, red and blue colors, which attract pollinators and seed dispersers and protect plants against various biotic and abiotic stresses. Anthocyanins can also help protect humans against various disorders and diseases (Zhang et al. 2014). Structural genes involved in anthocyanin biosynthesis and transcription factors (TFs) that control the transcription of these structural genes have been identified in various plants such as *Antirrhinum majus*, *Petunia*×*hybrida* (hereafter, petunia), *Zea mays*, and *Arabidopsis thaliana* (Davies et al. 2012; Koes et al. 2005). These factors include R2R3-MYB factors, basic helix-loop-helix (bHLH) factors, and WD40 factors, which form a complex called MBW (Albert et al. 2014; Baudry et al. 2004; Gonzalez et al. 2008; Koes et al. 2005; Xu et al. 2015). Structural genes of anthocyanin biosynthesis are grouped into early biosynthetic genes (EBGs) and late biosynthetic genes (LBGs), and the two groups are regulated differently (Lepiniec et al. 2006; Martin et al. 1991; Pelletier et al. 1997; Quattrocchio et al. 1993; Zhang et al. 2014). The MBW complex controls the transcription of LBGs (Lepiniec et al. 2006; Xu et al. 2015; Zhang et al. 2014). In addition, the MBW complex positively or negatively controls genes for TFs for anthocyanin biosynthesis including those that encode a protein included in the complex itself and thus respectively activating or repressing anthocyanin accumulation. Genes for TFs thus form a transcriptional network that involves positive and negative regulators (Albert et al. 2014; Xu et al. 2015).

In the petals of petunia, a model plant for the study of the genetics of anthocyanin biosynthesis (Vandenbussche et al. 2016), the structural genes involved in anthocyanin biosynthesis pathways and the TFs that regulate anthocyanin biosynthesis, especially those that function in the MBW complex, have been analyzed extensively (e.g., Albert et al. 2014; Koes et al. 2005). Genes known so far to encode TFs that activate anthocyanin biosynthesis in petunia include *anthocyanin* (*AN*) *2* (*AN2*), *AN4*, *DEEP PURPLE* (*DPL*) and *PURPLE HAZE* (*PHZ*) (encoding R2R3-MYB factors), *AN1* and *JAF13* (encoding bHLH factors), and *AN11* (encoding a WD40 factor). Two classes of MYB TFs that function as repressors have also been identified: *MYB27* (encoding an R2R3-MYB factor) and *MYBx* (encoding an R3-MYB factor) (Davies et al. 2012). While the gene encoding WD40 (*AN11*) is ubiquitously expressed in plant tissues (de Vetten et al. 1997), those encoding factors R2R3-MYB (e.g., *AN2*) and bHLH (*AN1* and *JAF13*) are expressed in a tissue-specific manner (Albert et al. 2011; Spelt et al. 2000; Zhang et al. 2019) and shape the pigmentation pattern of plant tissues. In particular, R2R3-MYB factors can be regarded as a determinant of pigmentation patterns through anthocyanin accumulation not only in petunia but also in a wide variety of plants (Davies et al. 2012). The R2R3-MYB and R3-MYB factors in petunia (AN2, AN4, DPL, PHZ, MYB27, and MYBx) have diversified in expression pattern and hence have distinct roles in terms of tissue-specific pigmentation and/or responses to environment. For example, AN2 controls pigmentation in petal limbs and AN4 in flower tubes and anthers (Quattrocchio et al. 1999; Spelt et al. 2000). DPL and PHZ control pigmentation associated with the petal veins and surface, respectively (Albert et al. 2011). Both *PHZ* and *MYBx* are highly expressed in high light, whereas *MYB27* is highly expressed in shade (Albert et al. 2011).

In petunia, anthocyanins can also accumulate in anthers. In contrast to the extensive information on pigmentation in flower petals, information on the process of pigmentation in anthers is limited even though some commercial varieties have pigmented anthers and others do not. Genetic analyses have shown that pigmentation of anthers in petunia is controlled by locus *AN4* (de Vlaming et al. 1984; Quattrocchio et al. 1993), which encodes an R2R3-MYB factor (Albert et al. 2011). Transgenic approaches have shown that the introduction of a gene encoding R2R3-MYB factor AN2 (Quattrocchio et al. 1998), DPL, PHZ (Albert et al. 2011), or AN4 (Zhang et al. 2021) induces pigmentation in anthers in petunia plants that are typically nonpigmented. These observations indicate that ectopic expression of genes encoding these TFs induces expression of structural genes for anthocyanin biosynthesis necessary for pigmentation in anthers of petunia. However, the mechanisms (e.g., cascades of transcriptional control and gene expression) involved in anther pigmentation have not been elucidated.

Here we addressed the mechanisms of anther pigmentation by producing transformants that ectopically express the *PHZ* gene in the *an4*^−^ genetic background and analyzing changes in regulatory networks and the expression of structural genes of anthocyanin biosynthesis that leads to pigmentation in anthers. We also comparatively analyzed gene expression profiles between varieties with pigmented anthers and those with nonpigmented anthers. Our data indicate that the TFs are functionally redundant and that establishing a state in which limited genes of anthocyanin biosynthesis, namely, the LBGs, are highly expressed is characteristic of anther pigmentation in petunia.

## Materials and methods

### Plant materials, plasmid construction and plant transformation

Petunia varieties Baccara Magenta (Magenta), Baccara Plum (Plum), and Baccara Blue ver. 2 (Blue) (Sakata Seeds) were used to analyze gene expression. Magenta produces nonpigmented anthers; Plum and Blue produce purple-pigmented anthers. Anthers were categorized into three developmental stages according to Koes et al. (1989) and used for RNA isolation: stage 1, bud size <15 mm; stage 2, bud size 15–30 mm; stage 3, bud size 30–40 mm. Transgenic petunia plants containing the coding region of the *PHZ* gene were produced using an inbred line V26. A DNA fragment containing the coding region of *PHZ* was amplified by PCR from cDNA synthesized from total RNA isolated from petal tissues of V26 line using KOD-Plus-Ver. 2 (TOYOBO). After being cloned into pGEM-T Easy vector (Promega), the DNA fragment containing the *PHZ* coding region was cloned between the cauliflower mosaic virus (CaMV) 35S promoter and nopaline synthase terminator in the pGreen 0029 vector (Hellens et al. 2000). Primers to construct plasmids are listed in Supplementary Table S1. The plasmid was introduced into *Rhizobium radiobacter* (syn. *Agrobacterium tumefaciens*) strain EHA105 cells that were resuspended in 20 mM CaCl_2_ by a freeze-thaw method (Holsters et al. 1978). The T-DNA region of the plasmid was then transferred to petunia essentially according to the method of Horsch et al. (1985) as follows. Fully grown leaves on petunia plants were harvested and surface-sterilized with 1.5% v/v sodium hypochlorite and 0.02% v/v Triton-X100, then washed with sterilized water. A slit was cut into each leaf with a scalpel and submerged for 2–3 days in a suspension of strain EHA105 cells grown overnight in YEP broth (5 g/L^−1^ yeast extract, 10 g l^−1^ Bacto Peptone, 5 g l^−1^ NaCl, pH 7.0). The leaves were then placed on MS agar (Murashige and Skoog 1962) containing 500 µg ml^−1^ cefotaxime, 50 µg ml^−1^ kanamycin, 1 µg ml^−1^ benzylaminopurine, 0.1 µg ml^−1^ naphthaleneacetic acid, 30 mg ml^−1^ sucrose and 0.8% agar to induce callus formation. Calli were subcultured on fresh medium every 10–14 days. Shoots that developed were excised from calli and transferred to hormone-free medium containing 500 µg ml^−1^ cefotaxime and 50 µg ml^−1^ kanamycin to induce rooting. After rooting, plantlets were transplanted to soil in pots, covered loosely with a plastic bag to maintain humidity and acclimated to the ambient environment for a week in a growth chamber. Plants were then grown in greenhouse or in the growth chamber with 16 h light/8 h dark at 24°C. Tissues of self-fertilized progeny were used for RNA isolation.

### Quantification of anthocyanins

Anthers and petals were taken from fully developed flower buds and opened flowers, respectively. These tissues were frozen with liquid nitrogen and ground to a powder with mortar and pestle. The powder was mixed with methanol containing 1% (v/v) HCl and incubated at room temperature for 48 h under dark. The solution was centrifuged at 2000×g for 1 min. Absorbance of the resulting supernatant was measured at 530 nm using NanoDrop 2000c spectrophotometer (NanoDrop Technologies) and was used to quantify anthocyanins. Quantification was done using cyanidin-3-*O*-glucoside as a standard. The extraction and quantification were performed in five biological replicates.

### RNA isolation and qRT-PCR

Total RNA was isolated from anther tissues essentially as described previously (Kanazawa et al. 2007; Kasai et al. 2012). The cDNA synthesis reaction mixture was prepared by mixing 4 µl of 5× reaction buffer (250 mM Tris-HCl [pH 8.3], 375 mM KCl, 15 mM MgCl_2_), 2 µl of 0.1 M dithiothreitol, 1 µl of 100 µM oligo(dT)20 primer, 4 µl of 2.5 mM dNTPs, the total RNA solution, and water to a final volume of 19 µl. The mixture was heated at 65°C for 5 min and rapidly cooled on ice. After the addition of 1 µl of reverse transcriptase M-MLV (Invitrogen), cDNA synthesis was performed at 42°C for 1 h. The reverse transcriptase was inactivated by heating the sample at 99°C for 1 min. Quantitative reverse transcription-PCR (qRT-PCR) was done using a 1-µl aliquot of the reaction mixture and TB Green Premix Ex Taq II (Tli RNaseH Plus) (Takara Bio) with CFX96 Real-Time System (Bio-Rad). The PCR cycle was 95°C for 30 sec, 58°C for 30 s, 72°C for 30 s, and 78°C for 2 s. This cycle was repeated 40 times. Fluorescence quantification was carried out before and after the incubation at 78°C to monitor for the formation of primer-dimers. A reaction mixture without reverse transcriptase was used as a control to confirm that no amplification occurred from genomic DNA contamination of the RNA sample. Amplification of a single DNA species was confirmed by melting curve analysis. Primers used to amplify DNA fragments are listed in Supplementary Table S1.

### Nucleotide sequence analysis

A DNA fragment that encompasses the coding region of *AN4* was amplified by PCR from genomic DNA using primers that were designed to anneal sequences conserved between *Petunia axillaris* and *Petunia inflata* upstream and downstream of the coding sequence. The PCR products were cloned into the pGEM-T Easy vector (Promega) and sequenced. The nucleotide sequences of PCR products were also analyzed by direct sequencing. Primers used for amplifying DNA fragments are listed in Supplementary Table S1. The nucleotide sequence data were deposited in the DDBJ database (accessions LC742841–LC742844).

### Statistical analyses

Differences in the anthocyanin levels between control plants and transgenic plants containing *PHZ* transgene were analyzed using the Mann–Whitney *U*-test. Differences in the mRNA levels between these plants were analyzed using the Steel test and those among different varieties were analyzed using the Steel–Dwass test.

## Results

### Ectopic expression of the *PHZ* gene induces changes in expression profiles of genes encoding TFs

**Figure 1:**
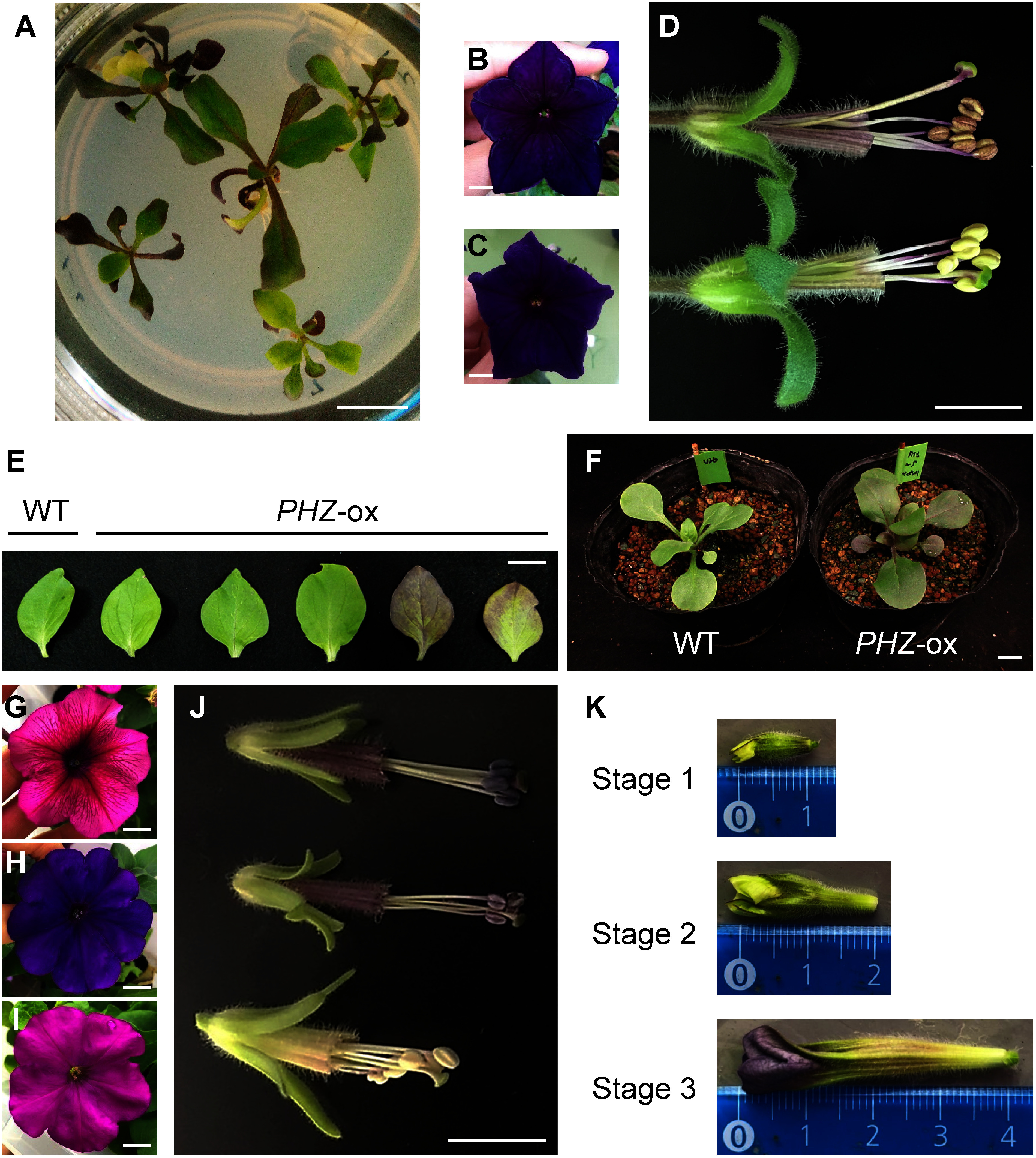
Figure 1. Phenotypes of petunia plants used in this study. (A–F) Transgenic plants harboring the *PHZ* transgene (*PHZ*-ox) and wild-type V26 plants. (A) Regenerated *PHZ*-ox plants grown in a medium after transformation. (B) Flower of *PHZ*-ox plants. (C) Flower of wild-type V26 plants. (D) Anther phenotypes of *PHZ*-ox (upper) and wild-type V26 (lower) plants, which were visualized by removing petal limbs from the flower. (E) Leaves of wild-type V26 and *PHZ*-ox plants. (F) Young plants of *PHZ*-ox (self-fertilized progeny) and wild-type V26. (G–J) Phenotypes of three varieties. Flowers of Plum (G), Blue (H) and Magenta (I). (J) Anther phenotypes of Plum (upper), Blue (middle) and Magenta (lower). (K) Flower buds of three developmental stages used for RNA isolation from anthers. Stage 1, bud size <15 mm; stage 2, bud size 15–30 mm; stage 3, bud size 30–40 mm. Scale bars = 1 cm.

**Figure 2:**
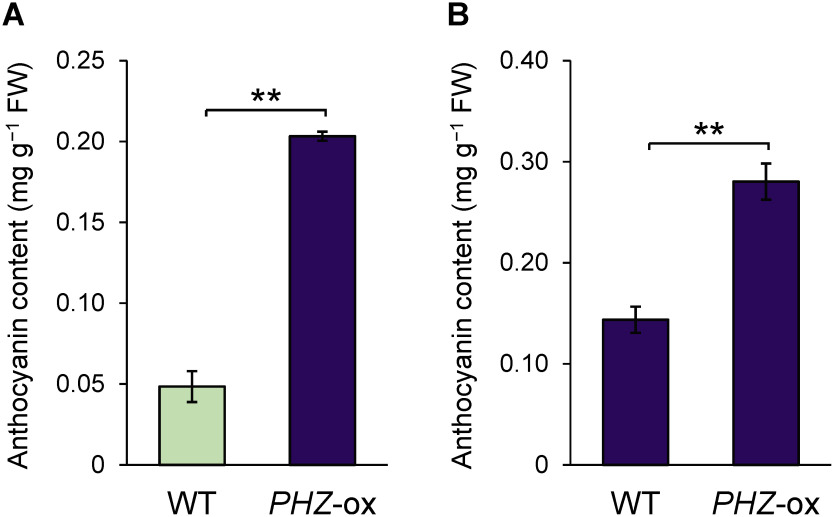
Figure 2. Changes in anthocyanin level as a consequence of the introduction of the *PHZ* transgene. Anthocyanin levels in anthers (A) and petals (B). *PHZ*-ox lines 13 and 2 were used for quantification in anthers and petals, respectively. Data are means and standard errors obtained from five biological replicates. Purple bars indicate pigmentation in respective tissues. ** *p*<0.01.

We produced transgenic petunia plants that contain a *PHZ* transgene(s) controlled by the CaMV 35S promoter (“*PHZ*-ox”) using plants of the V26 line that has the *an4*^−^ genotype and produces nonpigmented anthers. In plants regenerated from calli transformed with the *PHZ* transgene, various portions of vegetative tissues were pigmented (Figure 1A). While all 33 transformants produced flower petals whose color was identical to nontransgenic wild-type plants (Figure 1B, C), some of them produced partially pigmented leaves (Figure 1E) as was observed after regeneration, and all produced pigmented anthers (Figure 1D). In the subsequent generation of the *PHZ*-ox plants, the vegetative tissues were more evenly pigmented than those in the primary transformants (Figure 1F). Quantification of anthocyanins indicated that anthocyanins were accumulated at a higher level in the anthers of *PHZ*-ox plants than those of the wild type (Figure 2A). A higher level of anthocyanins was also detected in the petals of *PHZ*-ox plants (Figure 2B), although the petals were visually unchanged.

**Figure 3:**
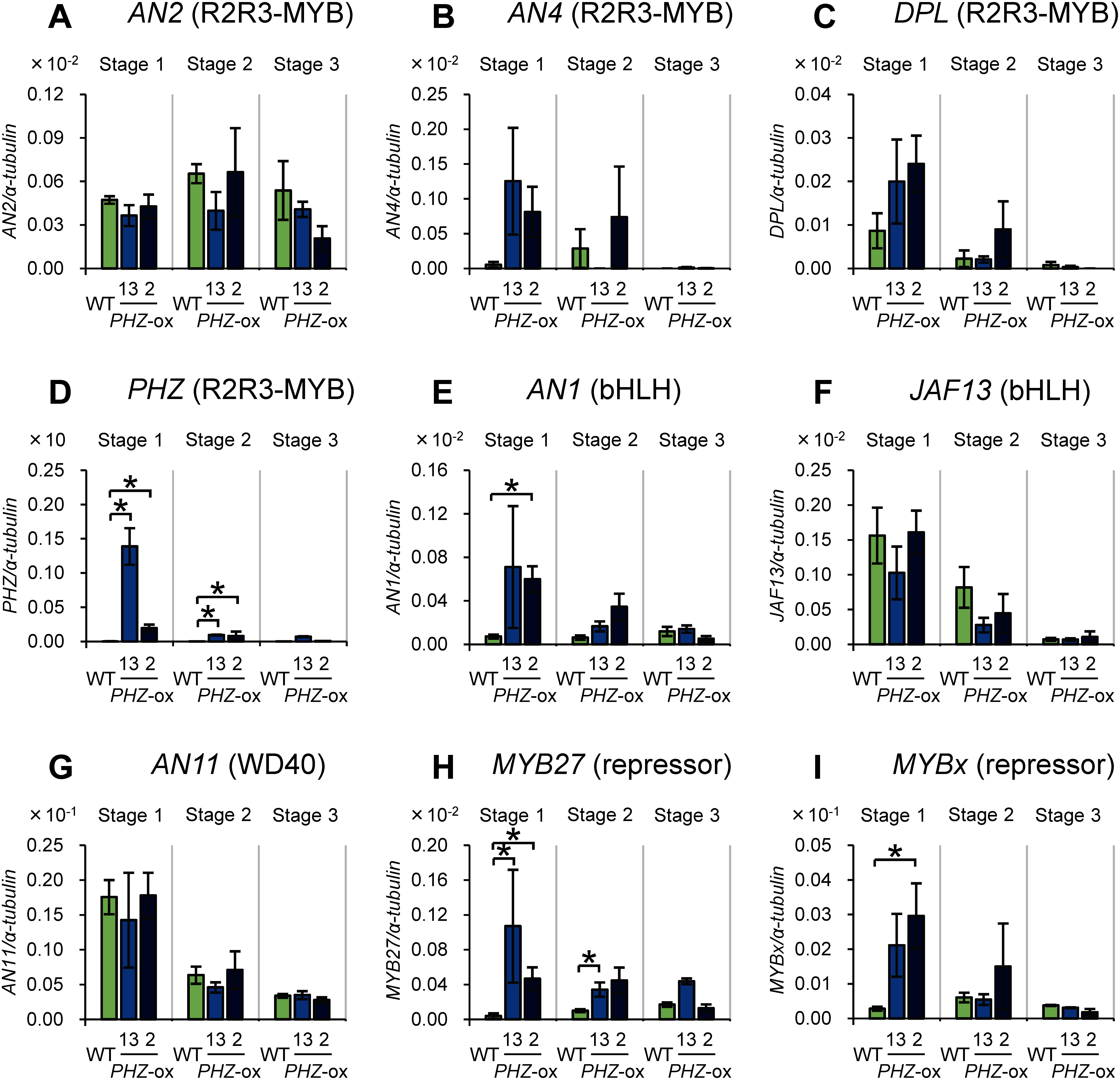
Figure 3. Changes in mRNA levels of genes encoding TFs involved in anthocyanin biosynthesis as a consequence of ectopic *PHZ* expression in anthers. (A–D) Genes encoding R2R3-MYB TFs: *AN2* (A), *AN4* (B), *DPL* (C) and *PHZ* (D). (E and F) Genes encoding bHLH TFs: *AN1* (E) and *JAF13* (F). (G) Gene encoding WD40: *AN11*. (H and I) Genes encoding repressors (R2R3-MYB and R3-MYB TFs): *MYB27* (H) and *MYBx* (I). Data for wild-type V26 plants (WT) and two independent transformants (lines 13 and 2 of *PHZ*-ox) at three developmental stages of anthers are shown. Experiments were done in five biological replicates. Data are means and standard errors. The α-*tubulin* gene was used as an internal control. Note that, in principle, values on the *y*-axis are comparable between experiments using the same primer sets but not between those using different primer sets (i.e., between the data of different panels in this figure). * *p*<0.05.

We analyzed the mRNA level of *PHZ* gene and those of the genes encoding TFs controlling structural genes involved in anthocyanin biosynthesis in the anthers of transformants. Genes involved in anthocyanin biosynthesis in anthers are expressed mainly in the early stages of flower development (Koes et al. 1989; Quattrocchio et al. 1993). We analyzed anthers of three early developmental stages that were classified previously (Koes et al. 1989; Figure 1K) in two plant lines that were independently transformed with the *PHZ* transgene (Figure 3).

We found that the mRNA level of *PHZ* gene was profoundly higher in stages 1 and 2 in the transformants (Figure 3D) than in the wild type. Among the genes encoding TFs that can function as an activator, the mRNA level of *AN1* was significantly higher in one of the transformants (Figure 3E). We also found that genes encoding two transcription factors that function as a repressor, MYB27 and MYBx, were upregulated in one or both of the transformants (Figure 3H, I). No statistically significant changes in the mRNA level were detected for the other TF genes that we analyzed at any stages of anther development (Figure 3).

### Changes in the expression of structural genes involved in anthocyanin biosynthesis

**Figure 4:**
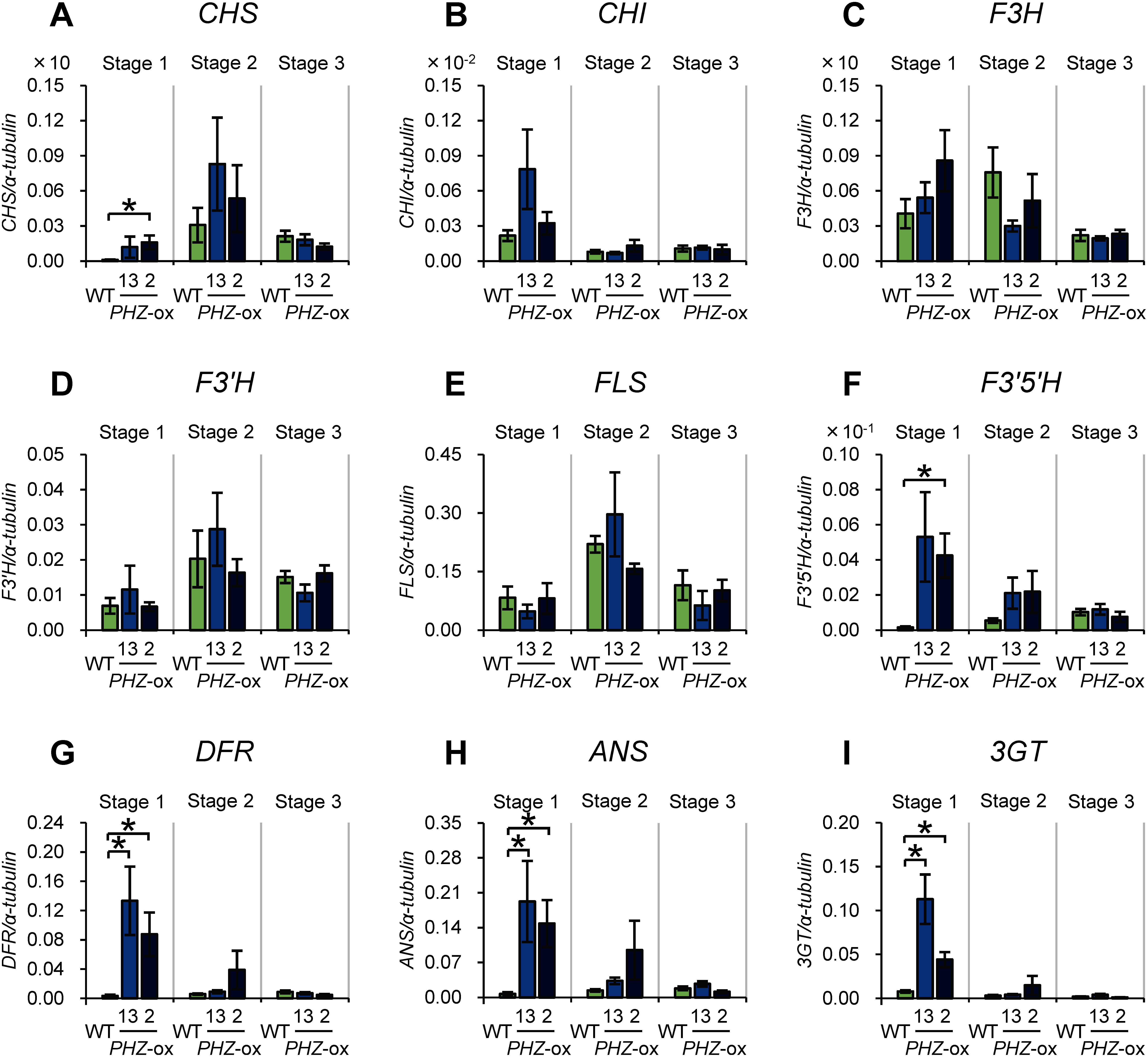
Figure 4. Changes in mRNA levels of structural genes of anthocyanin biosynthesis as a consequence of ectopic *PHZ* expression in anthers. (A–E) EBGs: *CHS* (A), *CHI* (B), *F3H* (C), *F3′H* (D) and *FLS* (E). (F–I) LBGs: *F3′5′H* (F), *DFR* (G), *ANS* (H) and *3GT* (I). For more information, see legend for Figure 3.

We next analyzed the mRNA levels of structural genes encoding enzymes involved in the biosynthesis of anthocyanins: chalcone synthase (CHS), chalcone isomerase (CHI), flavanone 3-hydroxylase (F3H), flavonoid 3′-hydroxylase (F3′H), flavonol synthase (FLS), flavonoid 3′,5′-hydroxylase (F3′5′H), dihydroflavonol 4-reductase (DFR), anthocyanidin synthase (ANS) and flavonoid 3-*O*-glucosyltransferase (3GT). Structural genes encoding enzymes involved in the biosynthesis of anthocyanins are grouped into two categories, EBGs and LBGs, in various plants (Martin et al. 1991; Pelletier et al. 1997; Quattrocchio et al. 1993), although categorization of genes into these groups may slightly vary between different species (Lepiniec et al. 2006; Zhang et al. 2014). In petunia, *CHS*, *CHI*, *F3H*, *F3′H*, and *FLS* are categorized into EBGs, and *F3′5′H*, *DFR*, and the genes for steps farther downstream in the pathway are categorized as LBGs (Lepiniec et al. 2006; Quattrocchio et al. 1993; Zhang et al. 2021; Supplementary Figure S1). The results of the qRT-PCRs indicated that the ectopic expression of *PHZ* did not affect the mRNA levels of the EBGs except for a slight increase of *CHS* (*CHS-A*) mRNA in a transformant (Figure 4A–E), whereas the mRNA levels of all four LBGs profoundly increased in one or both of the transformants (Figure 4F–I). Thus, the pigmentation of anthers induced by the ectopic expression of *PHZ* was accompanied by increased mRNA levels of LBGs in the transformants.

### Comparative analysis of varieties

To understand gene expression profiles characteristic of these different anther pigmentation phenotypes, we next comparatively analyzed two petunia varieties that produce pigmented anthers and one that produces nonpigmented anthers. Because anther pigmentation is controlled by the *AN4* gene, we first analyzed the nucleotide sequences of the coding region of *AN4* in the varieties and V26 (Supplementary Figure S2). V26 has a recessive allele (*an4*^−^) at this locus (Quattrocchio et al. 1993). Among the lines analyzed, we found some polymorphisms in the amino acid sequences encoded by *AN4*. Noteworthy is the finding that the coding sequence of *AN4* in V26, which produces nonpigmented anthers, was completely identical to that in V30 (*An4*^+^) line, which produces pigmented anthers (the V30 sequence was reported previously [HQ428105]; Albert et al. 2011). Thus, the absence of pigmentation in anthers is not ascribed to the coding sequence of *AN4*, but possibly to a low level of gene expression, at least in V26.

**Figure 5:**
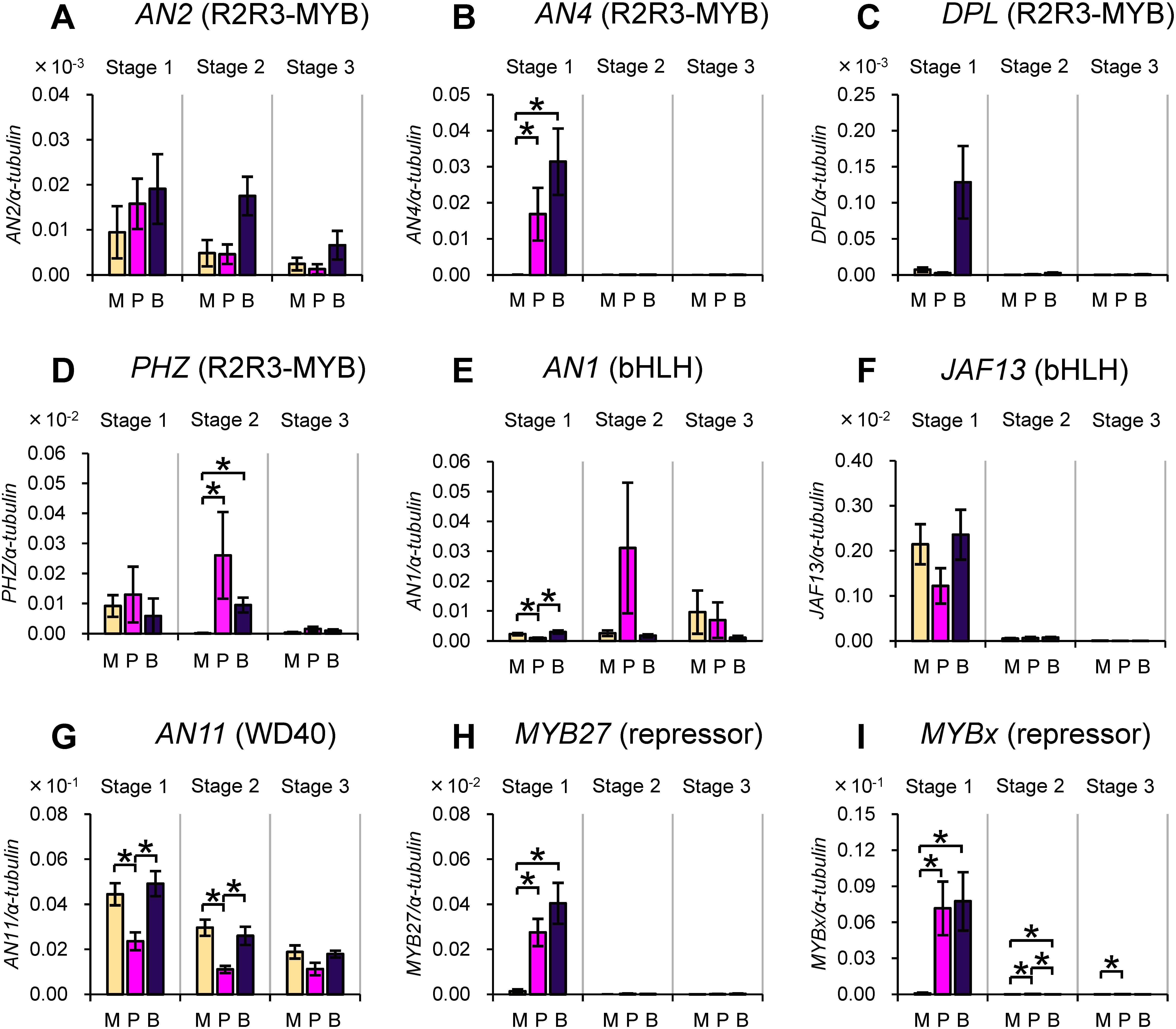
Figure 5. Differences in mRNA levels of genes encoding TFs involved in anthocyanin biosynthesis among varieties that produce pigmented anthers and nonpigmented anthers. (A–D) Genes encoding R2R3-MYB TFs: *AN2* (A), *AN4* (B), *DPL* (C) and *PHZ* (D). (E and F) Genes encoding bHLH TFs: *AN1* (E) and *JAF13* (F). (G) Gene encoding WD40: *AN11*. (H and I) Genes encoding repressors (R2R3-MYB and R3-MYB TFs): *MYB27* (H) and *MYBx* (I). Data for variety Magenta that produces nonpigmented anthers and Plum and Blue that produce pigmented anthers are shown. These varieties are abbreviated to M, P and B, respectively, in each panel. Experiments were done in five biological replicates. Data are means and standard errors. The α-*tubulin* gene was used as an internal control. Note that, in principle, values on the *y*-axis are comparable between experiments using the same primer sets but not between those using different primer sets (i.e., between the data of different panels in this figure). * *p*<0.05.

Anthers of the Plum and Blue varieties gradually started to become visibly pigmented in stage 2 in our growth conditions. (Regardless, we refer to the anthers of these varieties as “pigmented” at all stages.) The qRT-PCR analysis of genes encoding TFs indicated that transcripts of *AN4* were present at stage 1 of the pigmented anthers of the two varieties (Plum and Blue) but were barely detectable in the nonpigmented anthers (Magenta) (Figure 5B). *AN4* transcripts were not detected in anthers at stages 2 and 3 in any of the three varieties. The mRNA levels of the *MYB27* and *MYBx* genes were also higher in the pigmented anthers of both varieties (Plum and Blue) than in the nonpigmented anthers of Magenta at stage 1 of anther development (Figure 5H, I), coincident with the expression of *AN4*. Differences in the mRNA level were also detected for *PHZ*, *AN1* and *AN11*. The mRNA level of *PHZ* was higher in the pigmented anthers of Plum and Blue than in the nonpigmented anthers of Magenta in stage 2 (Figure 5D); differences in the mRNA levels of *AN1* and *AN11* genes between varieties were not correlated with pigmented/nonpigmented phenotypes of anthers (Figure 5E, G).

**Figure 6:**
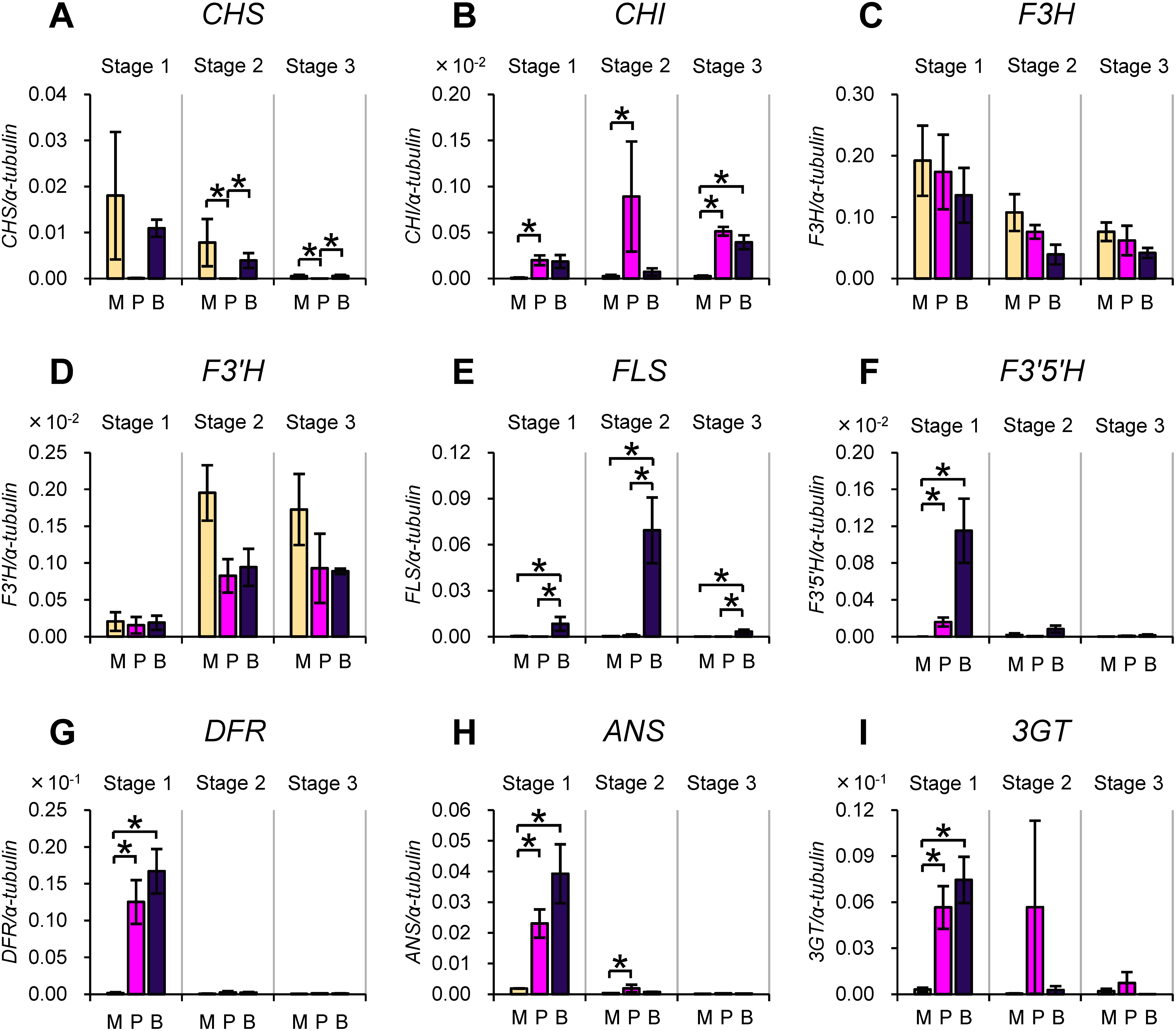
Figure 6. Differences in mRNA levels of structural genes of anthocyanin biosynthesis among varieties that produce pigmented anthers and nonpigmented anthers. (A–E) EBGs: *CHS* (A), *CHI* (B), *F3H* (C), *F3′H* (D) and *FLS* (E). (F–I) LBGs: *F3′5′H* (F), *DFR* (G), *ANS* (H) and *3GT* (I). For more information, see legend for Figure 5.

With regard to structural genes of anthocyanin biosynthesis (Figure 6A–E), differences in the mRNA levels between varieties were detected for the EBGs *CHS*, *CHI* and *FLS*, but the differences in the mRNA levels of *CHS* and *FLS* were not correlated with the pigmented/nonpigmented phenotypes of anthers (Figure 6A, E). Although the *CHI* mRNA level was higher in the pigmented anthers than nonpigmented anthers, this difference was detected at stage 3 (Figure 6B), after pigmentation had begun. In contrast, the mRNA levels of all four LBGs were higher in the pigmented anthers of Plum and Blue than in the nonpigmented anthers of Magenta at stage 1 (Figure 6F–I). In addition, the pattern of changes in the mRNA levels, namely, the mRNA accumulation in stage 1 and its subsequent decrease in stages 2 and 3, was commonly observed for the *AN4* gene and the four LBGs.

We also explored whether gene expression profiles may explain some of the characteristics of flower color in these varieties. Reduction in the *FLS* mRNA level results in an increase in anthocyanin production through altering a competitive balance between flavonol and anthocyanin production from common substrates (Davies et al. 2003; Holton et al. 1993). The *FLS* mRNA level was much higher in Blue than in Plum in anthers (Figure 6E) and the pigmentation of anthers was more intense in Plum than in Blue (Figure 1J). These observations suggest that the difference in the intensity of pigmentation in anthers between Blue and Plum is ascribed to the difference in the mRNA level of *FLS*.

We analyzed the mRNA levels of the *PHZ*, *CHS*, *F3′H*, *FLS* and *F3′5′H* genes in petals taken from fully developed flower buds before flower opening. We found that the relative mRNA levels of these genes among these varieties in petals (Supplementary Figure S3) were similar to those in anthers (Figures 5D, 6A, D–F), suggesting that relative relationships of gene expression between genes is essentially conserved between these tissues. A notable observation is that a high mRNA level of *F3′5′H* (Supplementary Figure S3), which encodes a protein that catalyzes production of delphinidin-based anthocyanins, was consistent with the purple color of petals in Blue (Figure 1H).

## Discussion

### Changes in the transcriptional regulatory networks as a consequence of ectopic *PHZ* expression

Here we presented a set of the profiles of changes in the mRNA levels of genes that encode structural genes and TFs involved in anthocyanin biosynthesis during anther development. We also showed how these profiles were modified as a consequence of the expression of a TF gene. Transgenic petunia plants containing the *PHZ* transgene controlled by the CaMV 35S promoter resulted in ectopic expression of *PHZ* in anthers where *PHZ* is intrinsically barely expressed. This expression induced pigmentation of anthers and concomitant changes in gene expression including those of genes encoding TFs. An increase in the mRNA level of a bHLH factor AN1, a key bHLH factor of the MBW activation complex (Albert et al. 2014), was observed. A previous study showed that a mutation of the *AN4* gene abolishes the expression of *AN1* in anthers, indicating that *AN1* expression depends on *AN4* (Spelt et al. 2000). Accordingly, the present results indicate that the ectopic expression of *PHZ* in the *an4*^−^ genetic background complemented the activity of *AN4*, at least its function to activate *AN1*.

PHZ protein directly binds to AN1 and JAF13 proteins and forms a complex (Albert et al. 2014). It is also known that *TT8*, which encodes a bHLH factor homologous to AN1 in *A. thaliana*, controls its own expression through a feedback regulation involving MBW complexes (Baudry et al. 2006; Xu et al. 2015, 2013). Considering these observations, a primary event induced by the ectopic *PHZ* expression is likely activation of *AN1* transcription, mediated by formation of the MBW complex including the PHZ protein and preexisting bHLH (e.g., AN1) and WD40 (e.g., AN11) factors. An increase in the level of *AN1* expression subsequently induces an increase in the formation of MBW complex comprising PHZ, AN1 and a WD40 protein, which can then enforce the transcriptional activation of *AN1* itself. In this scenario, the ectopic *PHZ* expression induces positive feedback, leading to the elevated formation of the MBW complex and elevated *AN1* expression.

Although PHZ also binds to another bHLH, JAF13 (Albert et al. 2014), the mRNA level of *JAF13* did not change, unlike that of *AN1*. This observation indicates a difference in the control of expression for *AN1* and *JAF13* (Spelt et al. 2000) and may reflect phylogenetic divergence between these genes (Davies et al. 2012; Quattrocchio et al. 1998). It is also known that *JAF13* does not compensate for the loss of *AN1* (Spelt et al. 2000).

Changes in mRNA level was also detected in *MYB27* (R2R3-MYB) and *MYBx* (R3-MYB), which encode repressors. Transcription of these genes is activated by the MBW complexes (Albert et al. 2014); hence, an increase in the expression levels of these genes can be explained by an increase in the expression levels of *PHZ* and *AN1*, which encode components of the MBW complex. Conversely, a previous study has shown that MYB27 is a component of the MBW complex, which represses the transcription of *AN1*, *MYB27* itself, and LBGs (Albert et al. 2014). Similarly, MYBx inhibits the ability of the MBW complex to activate structural genes by binding to AN1 (Albert et al. 2014). Therefore, ectopic *PHZ* expression could also induce negative control of the gene expression network of TFs through the activation of *MYB27* and *MYBx* as well. Thus, both the accelerator and the brake of anthocyanin biosynthesis were induced.

Ectopic *PHZ* expression induces an increase in the mRNA levels of *AN1*, *MYB27* and *MYBx* in leaf tissues of Mitchell petunia, with genotype combination *an2*^−^ *an4*^−^ *Dpl*^+^ *Phz*^+^ *An1*^+^ *An11*^+^ (Albert et al. 2014), and as we showed here, in the anthers of V26 (genotype combination *An2*^+^ *an4*^−^ *Dpl*^+^ *Phz*^+^ *An1*^+^ *An11*^+^). Presumably, a low expression level of R2R3-MYB factor(s) (e.g., AN2; Spelt et al. 2000) in both leaf and anther tissues might have allowed the manifestation of the function of the PHZ protein to activate these genes.

### Association between changes or differences in gene expression and anthocyanin pigmentation in anthers

Structural genes in the two categories, EBGs and LBGs, are distinct from each other in a coexpression network constructed on the basis of the “guilt-by-association” principle (Saito et al. 2008). Genes of these two categories are controlled by different TFs: in *A. thaliana*, EBGs are controlled by R2R3-MYB TFs, including MYB11, MYB12 and MYB111, while LBGs are controlled by the MBW complexes (Dubos et al. 2010; Stracke et al. 2007). In petunia, TFs for EBGs have not been identified. On the other hand, the control mechanisms of LBGs by the MBW complex is conserved in petunia, as exemplified by the finding that the mRNA levels of LBGs are markedly lower in petals of plants with a mutation in the *AN2*, *AN1* or *AN11* locus than in those that have dominant alleles at these loci, whereas the mRNA levels of EBGs are not affected by these mutations (Quattrocchio et al. 1993). A similar specific decrease in the mRNA levels of LBGs was also observed in anthers in plants that have a mutation in *AN4* (Quattrocchio et al. 1993).

Our data indicate that the mRNA levels of EBGs in anthers did not differ among the varieties or were not consistently correlated with pigmented vs nonpigmented phenotypes. In contrast, the expression levels of all four LBGs were profoundly higher in pigmented anthers of the transgenic (*PHZ*-ox) plants than in nonpigmented anthers of nontransgenic plants. The expression levels of LBGs were also higher in pigmented anthers of the two varieties (Plum and Blue) than in the nonpigmented anthers of Magenta. Intriguingly, the higher expression levels of the four LBGs were accompanied by higher expression levels of genes encoding TFs, MYB27 and MYBx, in the pigmented anthers of the *PHZ*-ox plants and the two varieties that express *AN4* (Plum and Blue). Supplementary Figure S4 shows the results of cluster analysis of genes using *k*-means algorithm on the basis of the ratios of mRNA levels in pigmented anthers vs nonpigmented anthers. As is evident in the cluster analysis, there is remarkable commonality between the genes that had higher mRNA levels associated with anther pigmentation detected in the *PHZ*-ox experiment (Supplementary Figure S4A) and those detected in the comparative analysis of existing varieties (Supplementary Figure S4B). This commonality suggests that the ectopic expression of *PHZ* changed the state of the transcriptional regulatory networks in the nonpigmented anthers to the state established in the pigmented anthers of existing varieties.

### Proposed model: Functional redundancy allows establishment of gene expression profiles that confer pigmentation in anthers

**Figure 7:**
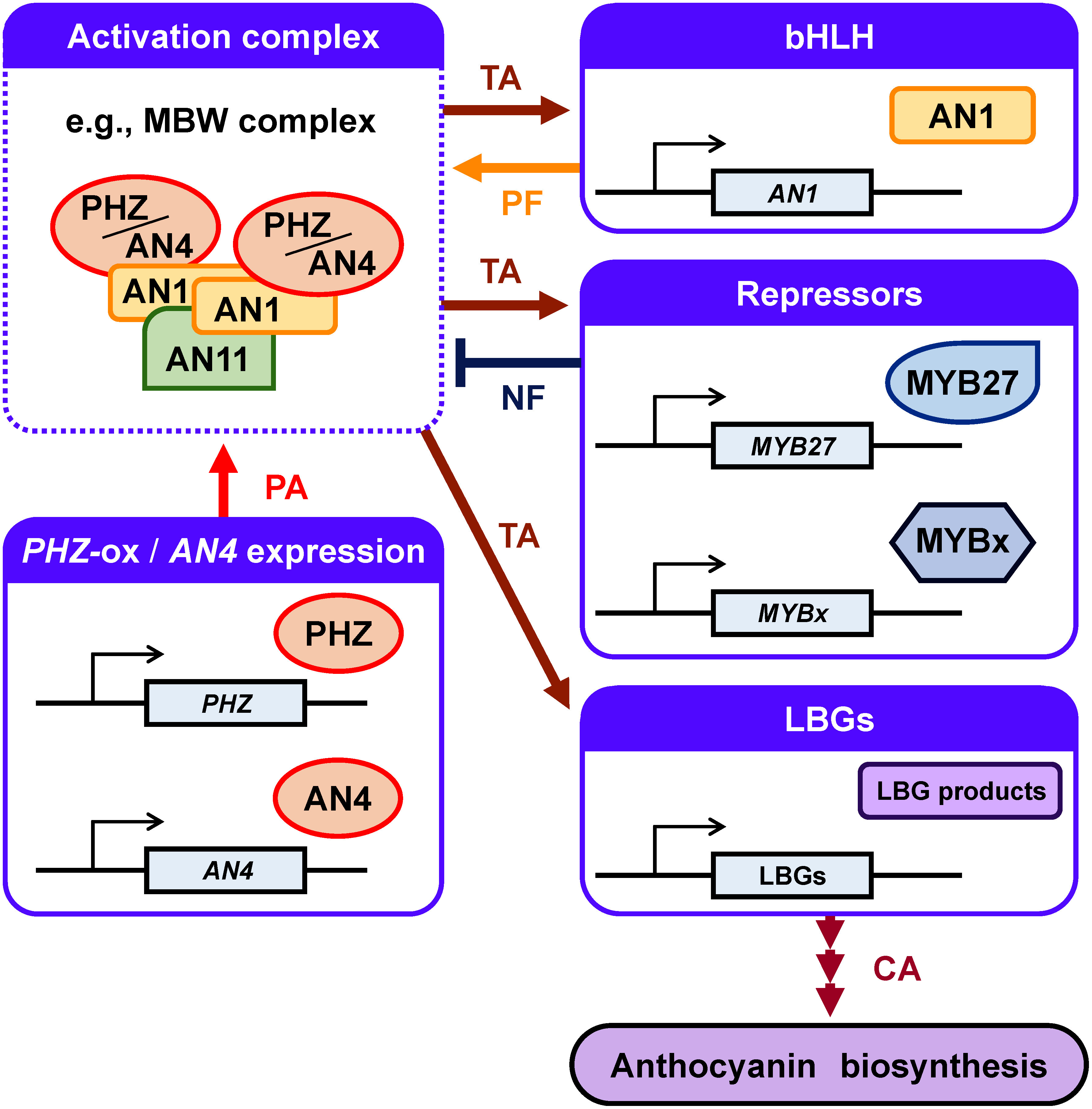
Figure 7. A model of transcriptional network that leads to anthocyanin production in anthers. Ectopic *PHZ* expression or the expression of the *AN4* gene in anthers induces transcriptional activation of TF genes, *MYB27* and *MYBx*, and LBGs. Ectopic *PHZ* expression also induces transcriptional activation of the *AN1* gene. Transcriptional activation of these genes is assumed to involve an activation complex such as the MBW complex. In nonpigmented anthers, LBGs are barely expressed so that anthocyanins are not produced, while their expression allows production of anthocyanins, thereby anthers become pigmented. In this model, an increase in the AN1 protein allows an increase in the activation complex via positive feedback. Conversely, increases in the mRNA levels of *MYB27* and/or *MYBx* lead to repression of the formation of an activation complex, constituting a negative feedback (see text). This process may prevent overproduction of anthocyanins. The diagram was prepared taking account of the conceptual frameworks illustrated in previous reports (Albert et al. 2014; Xu et al. 2015). Black arrows indicate transcription. PA, protein assembly; TA, transcriptional activation; PF, positive feedback; NF, negative feedback; CA, catalytic action.

Considering all our data regarding *PHZ*-ox plants and varieties with pigmented anthers and with nonpigmented anthers and the fact that the LBGs are structural genes for anthocyanin biosynthesis, we propose that the expression of LBGs at a high level is the characteristic state of pigmentation in anthers. In the two varieties that produce pigmented anthers, anther pigmentation was also correlated with higher expression of *AN4*, the key factor controlling anther pigmentation (de Vlaming et al. 1984; Quattrocchio et al. 1993). Our data suggest the following scenario. The state of the transcriptional network that allows the expression of LBGs at a high level is intrinsically established through the expression of *AN4* at a high level in anther-pigmented plants. Meanwhile, such a state can also be established by ectopic expression of another R2R3-MYB activator such as PHZ in plants that intrinsically produce nonpigmented anthers, which can substitute for AN4 as an activator in the MBW complex and lead to gene expression cascades necessary for pigmentation (Figure 7).

## Description of Supplementary Files

The supplementary materials for this article is available online.
