# Supplementary material for: Functional redundancy of R2R3-MYB transcription factors involved in anthocyanin biosynthesis is manifested in anther pigmentation in petunia
Source: Plant Biotechnol (Tokyo). 2024 Mar 25;41(1):9–18. doi: 10.5511/plantbiotechnology.23.1120a (PMC11500589; doi:10.5511/plantbiotechnology.23.1120a)
Supplement: Supplementary Data [file plantbiotechnology-41-1-23.1120a-s001.pdf]

Supplementary information

Functional redundancy of R2R3-MYB transcription factors involved in anthocyanin biosynthesis is manifested in anther pigmentation in petunia

Mashiro Yuhazu, Ryoko Hara, Mei Kimura, Akira Kanazawa

Supplementary Figures S1 to S4

Supplementary Tables S1

Supplementary Methods

Supplementary References

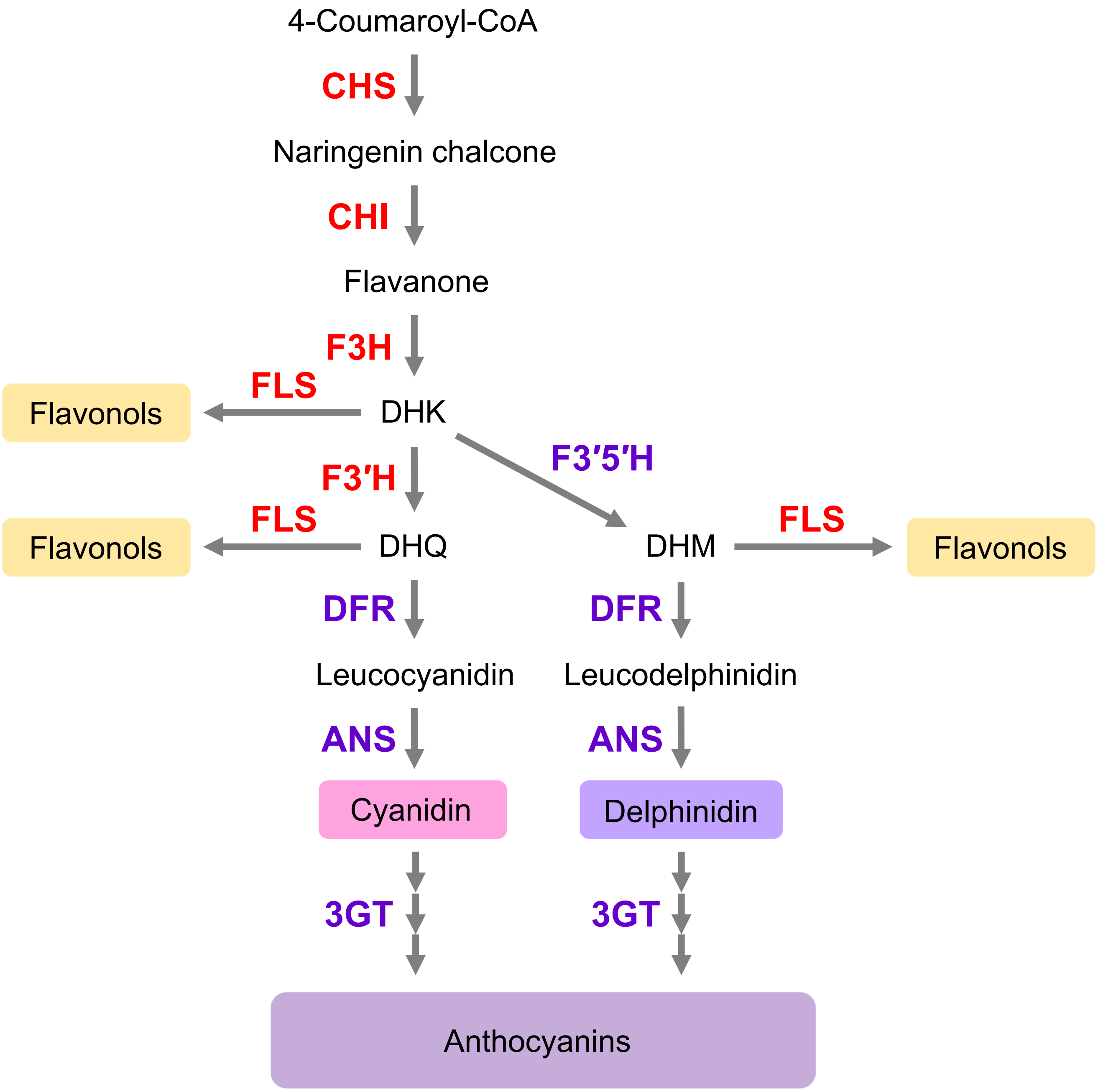

Figure S1. Biosynthetic pathways of anthocyanins in petunia. Enzymes encoded by EBGs are in red letters, those encoded by LBGs are in purple. CHS, chalcone synthase; CHI, chalcone isomerase; F3H, flavanone 3-hydroxylase; F3′H, flavonoid 3′-hydroxylase; FLS, flavonol synthase; F3′5′H, flavonoid 3′,5′-hydroxylase; DFR, dihydroflavonol 4-reductase; ANS, anthocyanidin synthase; 3GT, flavonoid 3-*O*-glucosyltransferase; DHK, dihydrokaempferol; DHQ, dihydroquercetin; DHM, dihydromyricetin. Note that petunia DFR does not accept DHK as substrate (Johnson et al. 2001).

```
                                                       R2
V30       MKTSVFTSSG VLRKGSWTEE EDILLRKCIE KYGEGKWHQV PVRAGLNRCR
V26       MKTSVFTSSG VLRKGSWTEE EDILLRKCIE KYGEGKWHQV PVRAGLNRCR
Plum      MKTSVFTSSG VLRKGSWTEE EDILLRKCIE KYGEGKWHQV PVRAGLNRCR
Blue      MKTSVFTSSG VLRKGSWTEE EDILLRKCIE KYGEGKWHQV PVRAGLNRCR
Magenta   MKTSVFTSSG VLRKGSWTEE EDILLRKCIE KYGEGKWHQV PVRAGLNRCR
          ********** ********** ********** ********** **********
                                                          R3
V30       KSCRLRWMNY LRPHIKRGDF SPDEVDLILR LHKLLGNRWS LIAGRLPGRT
V26       KSCRLRWMNY LRPHIKRGDF SPDEVDLILR LHKLLGNRWS LIAGRLPGRT
Plum      KSCRLRWMNY LRPHIKRGDF SPDEVDLILR LHKLLGNRWS LIAGRLPGRT
Blue      KSCRLRWMNY LRPHIKRGDF SPDEVDLILR LHKLLGNRWS LIAGRLPGRT
Magenta   KSCRLRWMNY LRPHIKRGDF SPDEVDLILR LHKLLGNRWS LIAGRLPGRT
          ********** ********** ********** ********** **********

V30       ANDVKNYWNT NLLRRSKFAP PQQHDRKCPK AIKTMAKNAI IRPQPRNLSK
V26       ANDVKNYWNT NLLRRSKFAP PQQHDRKCPK AIKTMAKNAI IRPQPRNLSK
Plum      ANDVKNYWNT NLLRRSKFAP PQQHDRKCPK AIKTMAKNTI IRPQPRNLSK
Blue      ANDVKNYWNT NLLRRSKFAP PQQHDRKCPK AIKTMAKNAI IRPQPRNLSK
Magenta   ANDVKNYWNT NLLTRSKFGP PQQHDRKCPK AIKTMAKNAI IRPQPWNLSK
          ********** *** **** * ********** ******** * ***** ****

V30       LAKNNVSTIH KDEHSKQEII IEKPTTAEVV SRDENVEWWT NLLLDNCNGF
V26       LAKNNVSTIH KDEHSKQEII IEKPTTAEVV SRDENVEWWT NLLLDNCNGF
Plum      LAKNNVSTIH KDEHSKQEII IEKPTTAEVV FRDENVEWWT NLLRDNSNGF
Blue      LAKNNVSTIH KDEHSKQEII IEKPTTAEVV SRDENVEWWT NLLLDNCNGF
Magenta   LAKNNVSTIH KDEHSKQEII IEKPTTAEVV SRDENVEWWT NLLLDNSNGF
          ********** ********** **********  ********* *** ** ***

V30       EKAATESTSA FKNIESLLNE ELLSPSINGG TYYPMQETRD MGWSDLSIDA
V26       EKAATESTSA FKNIESLLNE ELLSPSINGG TYYPMQETRD MGWSDLSIDA
Plum      EKAATESTSA FKNIESLLNE ELLSPSINGG TYYPMQETRE MGWSDLSIDA
Blue      EKAATESTSA FKNIESLLNE ELLSPSINGG TYYPMQETRD MGWSDLSIDA
Magenta   EKEATESTSA FQNIESLLNE ELLSPSINGG TYYPMQETGD MGWSDLSIDA
          ** ******* * ******** ********** ********   **********

V30       DLWELL
V26       DLWELL
Plum      DLWELL
Blue      DLWELL
Magenta   DLWELL
          ******
```

Figure S2. Alignment of amino acid sequences deduced from the nucleotide sequences of the *AN4* gene from V30 (HQ428105; Albert et al. 2011), V26 and varieties Plum, Blue and Magenta. Conserved amino acids are indicated by asterisks below the sequences. Amino acid residues present at a lower frequency in polymorphic sites are colored in red. The R2 and R3 MYB domains were assigned according to Stracke et al. (2001) and are indicated above the sequences.

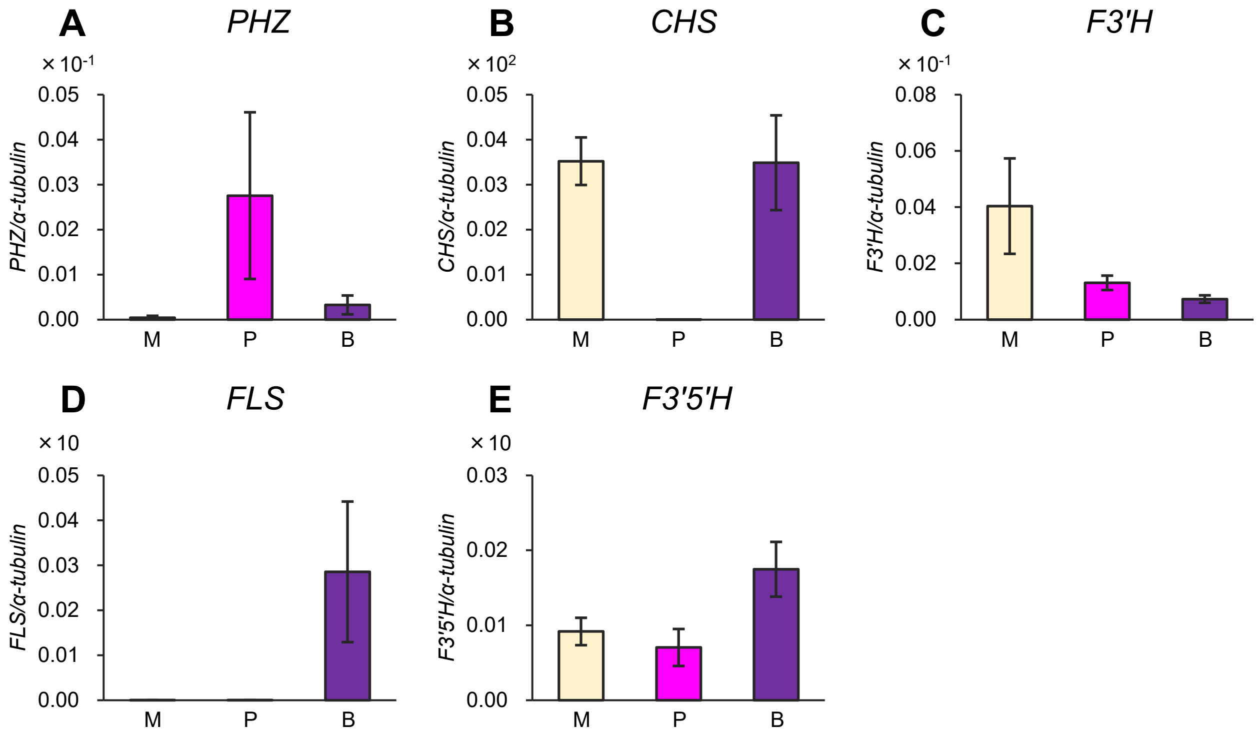

Figure S3. Differences in mRNA levels of genes involved in anthocyanin biosynthesis in petals among varieties that produce pigmented anthers and nonpigmented anthers. *PHZ* (A), *CHS* (B), *F3'H* (C), *FLS* (D) and *F3'5'H* (E). The *α-tubulin* gene was used as an internal control. M, Magenta; P, Plum; B, Blue. For related information, see legends for Figures 5 and 6.

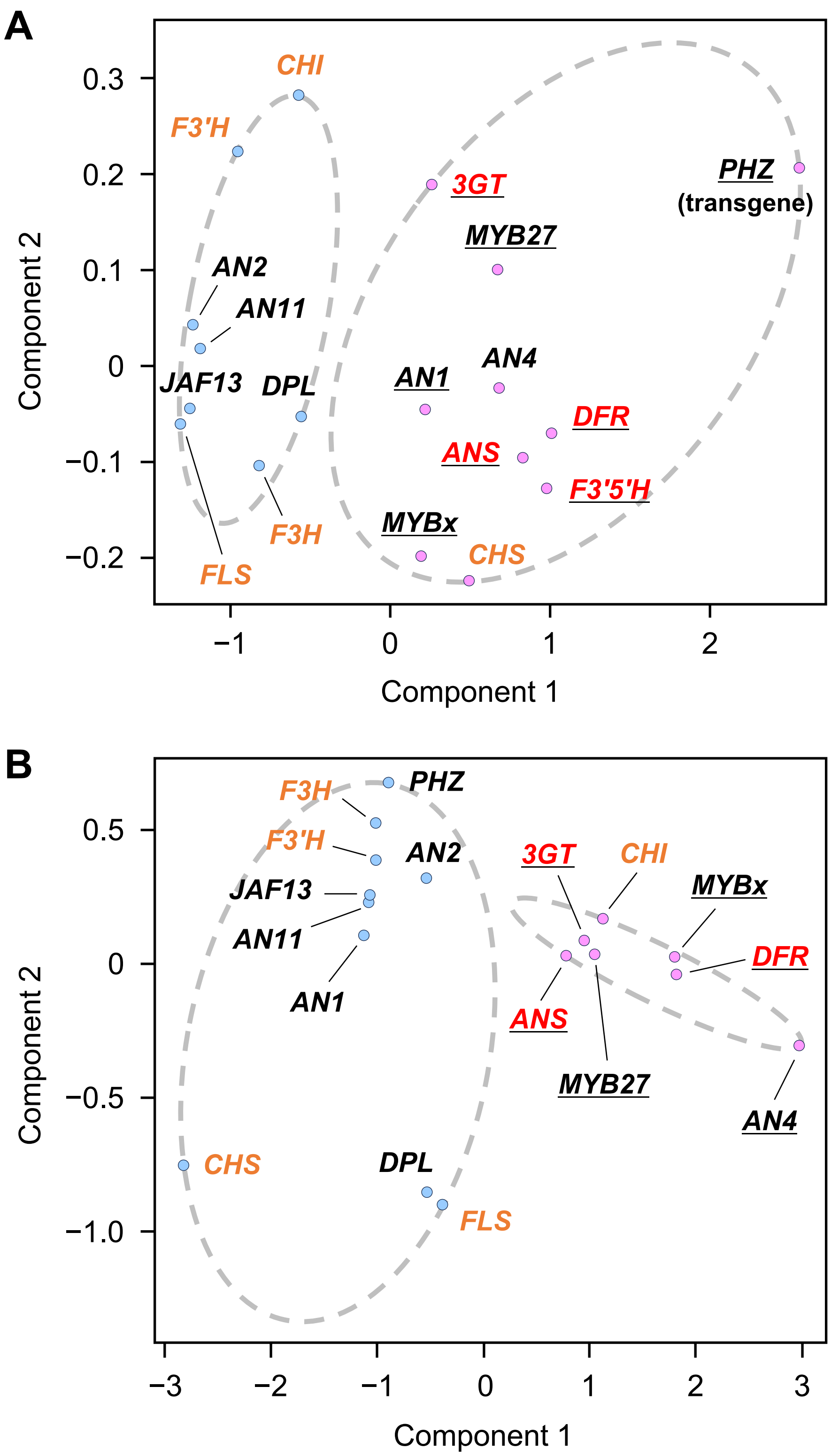

Figure S4. Cluster analysis of genes using *k*-means algorithm on the basis of the ratios of mRNA levels in pigmented anthers vs nonpigmented anthers. (A) *PHZ*-ox plants vs wild-type plants. (B) Plum and Blue vs Magenta. Genes encoding TFs, EBGs and LBGs are shown in black, orange and red letters, respectively. Genes whose higher mRNA levels were associated with anther pigmentation are underlined. Note that *AN4*, *MYB27*, *MYBx* and LBGs are grouped in the same cluster in both panels. The data of *F3'5'H* is missing in panel B because its mRNA level was below the detection limit of qRT-PCR in Magenta.

Table S1 List of PCR primers used in this study

Target gene	Primer name	Primer sequence	Accession No.
Primers for quantitative RT-PCR of genes encoding transcription factors			
AN2	Ph AN2 +227F<br>Ph AN2 +434R	5′-CTGGTCTGAATAGATGCAGG-3′<br>5′-CGAAGGTGGGTGTTCCAATA-3′	AF146702
AN4	Ph AN4 +812F<br>Ph AN4 +1010R	5′-TCCTCAGCAACACGATAGGA-3′<br>5′-AACGTTCTCGTCTCTGGACA-3′	HQ428105
DPL	Ph DPL + 988F<br>Ph DPL +1233R	5′-CAGTTAAGATCATGGCCAAG-3′<br>5′-CTGAAGTGCTTTCAGGTGCT-3′	HQ428107
PHZ	Ph PHZ +1764F<br>Ph PHZ +1921R	5′-CGGACCTTCTCAAATAGTGC-3′<br>5′-CACCATTGAACTCCATCGTC-3′	HQ428101
AN1 [a]	Ph AN1 +734F<br>Ph AN1 +1096R<br>Ph AN1 +1497F<br>Ph AN1 +1739R	5′-GGACTACACAACGGATTCAAG-3′<br>5′-CAAGCTGCATAAGTTCACTGG-3′<br>5′-GTGGCTGCTCAAAAGCATAC-3′<br>5′-ACAAAAGGAACAAGTGACCTC-3′	AF260919
JAF13	Ph JAF13 +8F<br>Ph JAF13 +126R	5′-GGGATGCAAAGACCATAATG-3′<br>5′-CCCTGGTTGTGAAACTGTAG-3′	AF020545
AN11	Ph AN11 ts +543F<br>Ph AN11 ts +815R	5′-GGCGTCGTTGAAACTCAGTT-3′<br>5′-CATAGCAGGCGACCTAATATC-3′	U94748
MYB27	Ph MYB27 +103F<br>Ph MYB27 +256R	5′-GGTTGCTGGCGAAATTTACC-3′<br>5′-CAAGTAGGGCATGAAGCTTG-3′	KF985023
MYBx	Ph MYBx +30F<br>PhMYBx +193R	5′-TGTAAATACTCCGGCTGATTC-3′<br>5′-TTCTTCCAGCTATAAGAGACC-3′	KF985022
Primers for quantitative RT-PCR of structural genes and internal control gene *α-tubulin*			
CHS	CHS +2150F<br>2349	5′-TCAAAGATGTTCCTGGGCTG-3′<br>5′-CCCTTCACCAGTAGTTCCTA-3′	AF233638
CHI	CHI 147F<br>CHI 286R	5′-TACGGCGATAGGTGTGTATC-3′<br>5′-GGCAAGATCGTAGTAACTCG-3′	AF233637
F3H	F3H 1600F<br>F3H 1955R	5′-ACTTGGATCACTGTTCAGCC-3′<br>5′-ATACACTATCGCCTCTGGTG-3′	AF022142
F3'H	F3'H +218F<br>F3'H +445R	5′-GCATCGGTTGCAGCTCAGT-3′<br>5′-GGACATGGCGGAAGTCATC-3′	AF155332
FLS	FLS +263F<br>FLS +399R	5′-GCATTCCTGATGAGGCTATC-3′<br>5′-CTGCAGAGAAGTTCCATAGC-3′	Z22543
F3'5'H	F3'5'H +218F<br>F3'5'H +384R	5′-GAACATGTGGCATGGCAGTT-3′<br>5′-TAGCAACTTCCATCGTGGTC-3′	Z22545
DFR	DFR 879F<br>DFR 952R	5′-GCTATCATCTACGATGTGGC-3′<br>5′-TGTCGACAAGTATCGATGGC-3′	AF233639
ANS	ANS 1006F<br>ANS 1031R	5′-TACCTGAGACTGTCACTGAG-3′<br>5′-GCAGTATCCAGTTCATCCTC-3′	X70786
3GT	3GT 873F<br>3GT 1069R	5′-GCAGTGGCAGAAGCATTAGA-3′<br>5′-CACATGATATGCCCTCCAAA-3′	AB027454
α-tubulin	Ph tub 125F<br>Ph tub 267R	5′-CAACTATCAGCCACCAACTG-3′<br>5′-CACGCTTGGCATACATCAGA-3′	LC782009
Primers for cloning DNA fragments and sequencing analysis			
PHZ	NcoI-PHZ -28F<br>BamHI-PHZ +2080R	5′-CCATGGGAGTCATATGCAGTGTGACC-3′<br>5′-GGATCCGGGAGAGAATAGGATCGACT-3′	
AN4	AN4 -141F<br>AN4 +1F<br>AN4 +1223R<br>AN4 +1283R	5′-TTAATGTTGTCCACGTTAGTTG-3′<br>5′-ATGAAAACTTCTGTTTTTACGTC-3′<br>5′-TCATTATAGTAATTCCCAGAGG-3′<br>5′-GAGATTACGTCACAATTACAAG-3′	

[a] Primers Ph AN1 +734F and Ph AN1 +1096R were used for V26 line and its transformants; primers Ph AN1 +1497F and Ph AN1 +1739R were used for varieties Magenta, Plum and Blue.

Supplementary Methods

Cluster analysis using the **k***-means algorithm*

K-means clustering of genes was done on the basis of the ratios of mRNA levels in pigmented anthers vs those in nonpigmented anthers. Data of mRNA levels in stage 1 of anther development were used. For each gene, the ratios of the average mRNA levels in *PHZ*-ox plants vs nontransgenic plants and those in Plum and Blue vs Magenta were calculated, respectively. These ratios were log-transformed and used for cluster analysis. Cluster analysis was done using *k*-means algorithm in the R statistical computing environment.

Supplementary References

Albert NW, Lewis DH, Zhang H, Schwinn KE, Jameson PE, Davies KM (2011) Members of an R2R3-MYB transcription factor family in *Petunia* are developmentally and environmentally regulated to control complex floral and vegetative pigmentation patterning. *Plant J* 65: 771-784

Johnson ET, Ryu S, Yi H, Shin B, Cheong H, Choi G (2001) Alteration of a single amino acid changes the substrate specificity of dihydroflavonol 4-reductase. *Plant J* 25: 325-333

Stracke R, Werber M, Weisshaar B (2001) The *R2R3-MYB* gene family in *Arabidopsis thaliana*. *Curr Opin Plant Biol* 4: 447-456
